# Structural Characterization of Al_0.37_In_0.63_N/AlN/p-Si (111) Heterojunctions Grown by RF Sputtering for Solar Cell Applications

**DOI:** 10.3390/ma14092236

**Published:** 2021-04-27

**Authors:** Arántzazu Núñez-Cascajero, Fernando B. Naranjo, María de la Mata, Sergio I. Molina

**Affiliations:** 1 Departamento de Electrónica, Universidad de Alcalá, GRIFO, 28871 Alcalá de Henares, Spain; arnunezc@ing.uc3m.es; 2 Departamento de Tecnología Electrónica, Universidad Carlos III de Madrid, 28911 Leganés, Spain; 3 Departamento de Ciencias de los Materiales e Ingeniería Metalúrgica y Química Inorganica, Universidad de Cádiz, IMEYMAT, 11510 Cádiz, Spain; maria.delamata@uca.es (M.d.l.M.); sergio.molina@uca.es (S.I.M.)

**Keywords:** III-nitrides, AlInN, AlN buffer, RF sputtering, TEM

## Abstract

Compact Al_0.37_In_0.63_N layers were grown by radiofrequency sputtering on bare and 15 nm-thick AlN-buffered Si (111) substrates. The crystalline quality of the AlInN layers was studied by high-resolution X-ray diffraction measurements and transmission electron microscopy. Both techniques show an improvement of the structural properties when the AlInN layer is grown on a 15 nm-thick AlN buffer. The layer grown on bare silicon exhibits a thin amorphous interfacial layer between the substrate and the AlInN, which is not present in the layer grown on the AlN buffer layer. A reduction of the density of defects is also observed in the layer grown on the AlN buffer.

## 1. Introduction

The ternary alloy, AlInN, has recently attracted the attention of researchers, because its tunable wide-range bandgap allows for its use in electronic, optoelectronic, and photonic applications [1,2,3,4,5]. However, it is difficult to grow single-phase AlInN due to its large immiscibility gap [6,7], which is caused by the different bonding energy [8], lattice parameter, growth, and decomposition temperatures [9] of the binaries (AlN and InN).

Different techniques have been used to grow AlInN, such as metal-organic chemical vapor deposition (MOCVD) [10,11,12], molecular beam epitaxy (MBE) [13,14,15,16], and the sputtering technique [17,18,19,20,21,22]. Unlike MOCVD or MBE, the sputtering technique uses an electrical discharge to extract the target species, where the generated ions and atoms are provided with kinetic energy by the sputtering process itself, thus overcoming the phase separation issues related to the heating procedures. The use of the sputtering technique allows for the deposition of the desired material on large substrates at a wide range of temperatures, resulting in polycrystalline layers.

Previous studies on the growth of AlInN on an AlN buffer using the sputtering technique [22,23] have been conducted; however, as far as we know, none of them have deeply studied the effect of an AlN buffer on the structural properties of AlInN or the accommodation mechanism.

In this work, the effect of using an AlN buffer layer on Si (111) substrates in terms of the structural properties of the subsequently sputtered AlInN layer are studied by high-resolution X ray diffraction measurements (HRXRD) and transmission electron microscopy (TEM).

## 2. Materials and Methods

The AlN and AlInN layers were grown on p-Si(111) substrates by radiofrequency (RF) sputtering using an ATC ORION 3 HV AJA International system (AJA International, Scituate, MA, USA). The system was equipped with separate 2” magnetron targets, one of pure In (4N5) and another of pure Al (5N).

The growth process was as follows: first, the targets were pre-sputtered with Ar (6N); meanwhile, the substrates were chemically cleaned in organic solvents and blown down with nitrogen. Once a base pressure of 10^−6^ Pa was achieved, the substrates were loaded into the growth chamber, and they were degassed for 30 min at 550 °C and pre-sputtered with Ar. Then, the substrate was cooled down to the growth temperature (450 °C), film deposition was conducted by introducing 6 sccm of pure N2 (6N), and the pressure was increased to 0.47 Pa. The AlN layer was grown by applying an RF power of 225 W to the Al target and the Al_0.37_In_0.63_N by co-sputtering the In (30 W) and Al (150 W) targets. The AlN layer thickness varied from 0 to 15 nm, while the Al_0.37_In_0.63_N was fixed at 80 nm. The substrate-target distance was fixed to 10.5 cm. The optimization of the growth of the AlN and Al_0.37_In_0.63_N layers can be found elsewhere [19,22]. It should be pointed out that the Al-content represents a suitable trade-off between electrical properties (the carrier concentration) and band gap energy for solar cell development [24].

The structural properties of the layers were studied by high-resolution X ray diffraction measurements using a PANalytical X’Pert Pro MRD system and by transmission electron microscopy using a JEOL2100 TEM microscope (JEOL Ltd., Akishima, Tokyo, Japan), equipped with a LaB_6_ gun operated at 200 kV. The chemical analyses performed by means of electron energy-loss spectroscopy (EELS) were carried out in scanning mode (STEM) at 200 kV using a JEOL2010F microscope (JEOL Ltd., Akishima, Tokyo, Japan), with a field-emission gun and a GATAN GIF energy filter (GATAN Inc., Pleasanton, CA, USA). The EELS experiments were conducted using a 0.3 eV energy dispersion at a camera length of 10 mm. The acquired EELS spectra were denoised using Principal Component Analysis (PCA) routines by choosing the suitable number of components in each case, before extracting the signals. 

## 3. Results and Discussion

**Figure 1:**
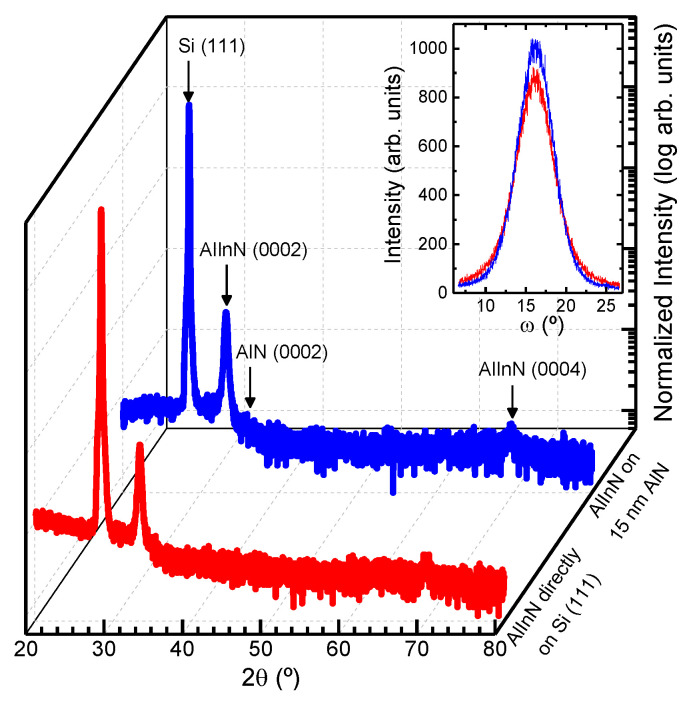
2θ/ω scans of the 80 nm-thick AlInN layers grown on bare silicon (111) (red) and on a 15 nm AlN buffer layer (blue). Inset: Rocking curves of the samples.

Figure 1 shows 2θ/ω scans of the 80 nm-thick AlInN layers grown on bare Si (111) and on a 15 nm AlN buffer layer on a Si (111) substrate. These scans are used to study the role of the initial growth surface in the lattice mismatch accommodation between nitride and silicon. They present a poly-crystalline wurtzite structure aligned along the c axis, with no phase separation. Only the substrate diffraction peak ((111) Si), the (0002) AlN, and the (0002) and (0004) AlInN diffraction peaks are shown. A reduction of the FWHM of the (0002) AlInN rocking curve from 5.6 to 5.3° is observed for the 15 nm-thick AlN buffer layer, indicating an improvement of the crystalline quality of the AlInN layer (see inset Figure 1).

**Figure 2:**
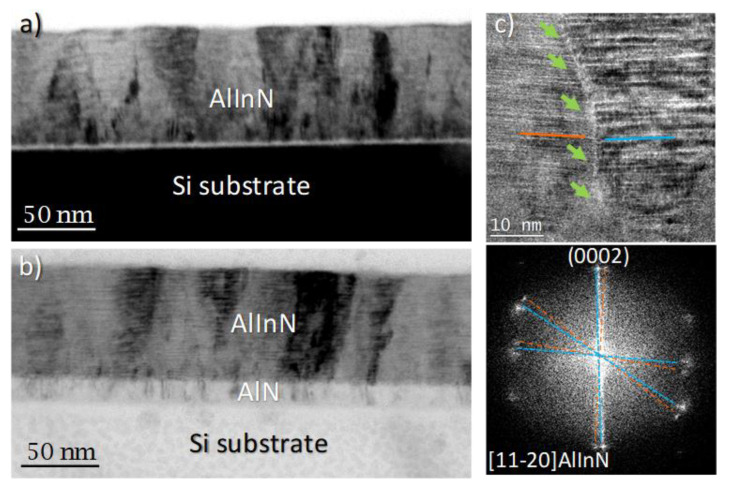
Diffraction contrast TEM images of: (**a**) AlInN grown on bare Si and (**b**) AlInN grown on 15 nm AlN. HRTEM details of a grain boundary within the AlInN layer is shown in (**c**), along with its fast Fourier transform (FFT), highlighting the misalignment between neighboring grains (blue and orange lines in (**c**) and FFT).

A deeper study of the structural properties was carried out using TEM measurements (JEOL Ltd., Akishima, Tokyo, Japan). First, the samples were studied under diffraction contrast conditions to show the structural defects over large areas (see Figure 2). The defect contrast is enhanced so that the structural defects appear as dark areas across the AlInN and AlN layers, propagating along the growth direction (Figure 2a,b). The density of defects was estimated from phase-contrast TEM images affected by crystal distortions, allowing for the quantification of the defects within the analyzed (known) layer area. This analysis indicates a reduction of one order of magnitude (from 10^11^ cm^−2^ to 10^10^ cm^−2^) for the 15 nm AlN buffer layer. The high lattice mismatch between the nitrides and Si (111) substrates, with in-plane lattice constants of a = 3.112 Å, a = 3.548 Å, and a = 3.840 Å, for AlN, InN, and Si (111), respectively, should also be noted [9]. High-resolution TEM (HRTEM) images show that the structural defects are mainly the grain boundaries nucleated at the interface (see Figure 2c), propagating through the film and associated dislocations. A similar reduction was obtained in the ternary nitrides (AlGaN) and GaN binary when introducing an AlN buffer grown by MBE [25,26].

**Figure 3:**
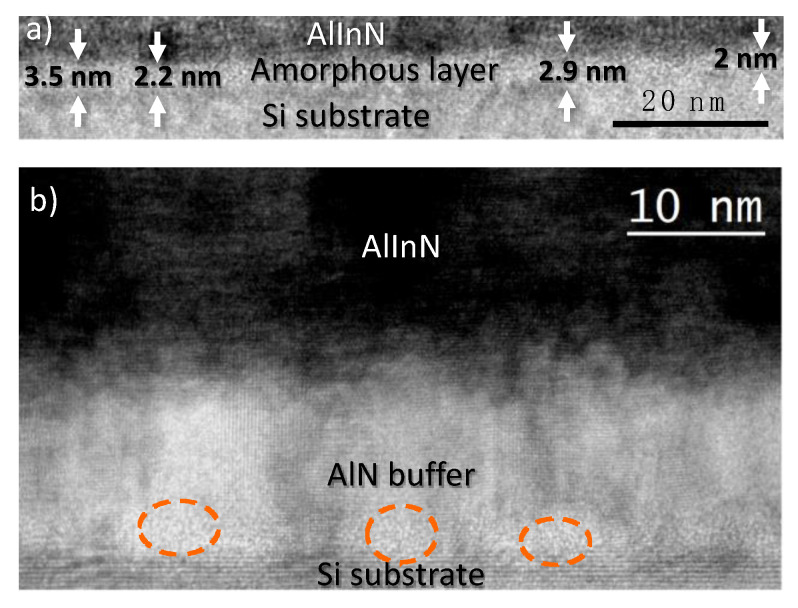
HRTEM zoom of the layer/substrate interface of: (**a**) the AlInN grown on bare silicon, and (**b**) the AlInN grown on 15 nm AlN.

The HRTEM measurements also show an amorphous layer of 2–3 nm-thick (see Figure 3a) in the case of the AlInN grown directly on bare silicon. However, with the inclusion of a 15 nm AlN buffer layer, this layer disappears, although small domains with quasi-amorphous AlN are observed (see Figure 3b). One possible explanation is that a higher RF power is applied to the Al target for the growth of AlN, compared to that applied for the growth of AlInN (225 W and 150 W, respectively). The higher kinetic energy of the incoming species when growing AlN facilitates the nucleation of the polycrystalline layer in the first stages of the deposition process. This effect is in accordance with the suitability of sputtering for growing AlN layers using a moderate substrate temperature (450 °C). 

**Figure 4:**
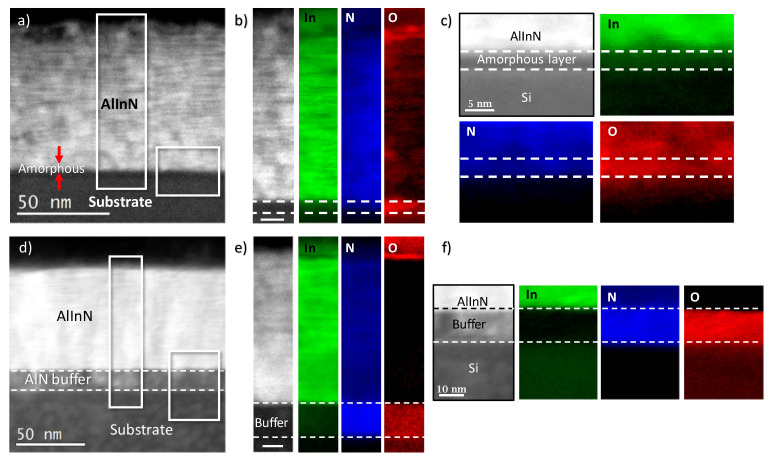
EELS analysis of the AlInN grown on (**a**–**c**) bare Si (**d**–**f**) using an AlN buffer. The figure displays the In, N, and O EELS signals mapped at the regions indicated in (**a**,**d**). The scale bar in (**b**,**e**) is 10 nm.

We performed further chemical analyses by means of electron energy-loss spectroscopy (EELS) to elucidate the nature of this amorphous layer (see Figure 4a–c)). The analysis reports the presence of nitrogen and oxygen at the interface between the substrate and the AlInN layer, which is in agreement with the formation of an interfacial amorphous SiOxNy layer. Importantly, oxygen is not only present in the amorphous layer, but also in the AlInN growing material, when using bare silicon as the substrate. In contrast, the oxygen content in the AlInN grown on AlN buffer layers shows a remarkable reduction, while the AlN buffer is highly oxygenated (Figure 4d–f). Therefore, it is likely that the AlN buffer traps the oxygen through the formation of Al-O bonds, thus hindering its diffusion into the AlInN growing layer, which is also the case for the AlInN layers deposited directly on Si (111). The O contamination may have come from the Si surface before growing, as due to the system limitations, it was degassed at a temperature below that required to remove the native oxide [27]. 

**Figure 5:**
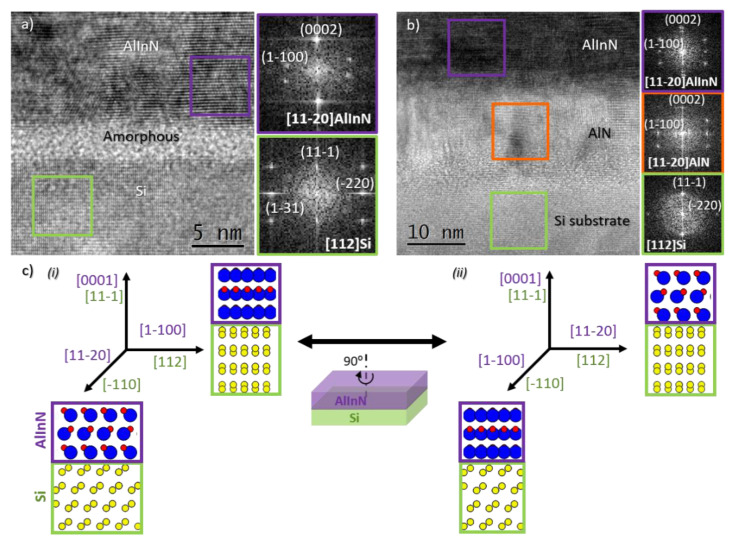
HRTEM images and indexed FFTs of: (**a**) AlInN grown on bare Si and (**b**) AlInN grown on a 15 nm AlN buffer layer. (**c**) Sketch showing two possible epitaxial relationships for the AlInN/Si system, including the most commonly reported alignment between the materials (i) and the actual epitaxial relationship observed in this study (ii).

The structural characterization carried out through HRTEM analyses reveal the epitaxial relationship, which is understood as the alignment between the main planes and directions of the growing AlInN and the silicon substrate, in both cases (with and without the buffer layer). Figure 5 shows the interatomic plane alignment between the AlInN layers and the underlying Si substrate, evidencing the following epitaxial relationship: (0001)[11$\bar{2}$0] AlInN||(11$\bar{1}$)[112] Si. It is also shown that in the sample grown on the AlN buffer layer, the AlInN and the AlN layers share an orientation and growth direction, as expected. This epitaxial relationship implies a 90° in-plane rotation of the AlInN layer, compared to the most common epitaxial relationship reported for III-nitrides grown on Si(111) (see Figure 5c), where the materials are usually related as follows: (0001)[11$\bar{2}$0] AlInN||(111)[1$\bar{1}$0] Si [28]. Our observation of another epitaxial relationship may be related to the existence of an initial amorphous layer on top of the silicon substrate. In this sense, Serban et al. observed the random in-plane orientation of AlInN nanostructures deposited by RF sputtering, which evolves into the commonly obtained orientation when using the HF cleaning of Si substrates, before the deposition of the nitride layers [29]. 

**Figure 6:**
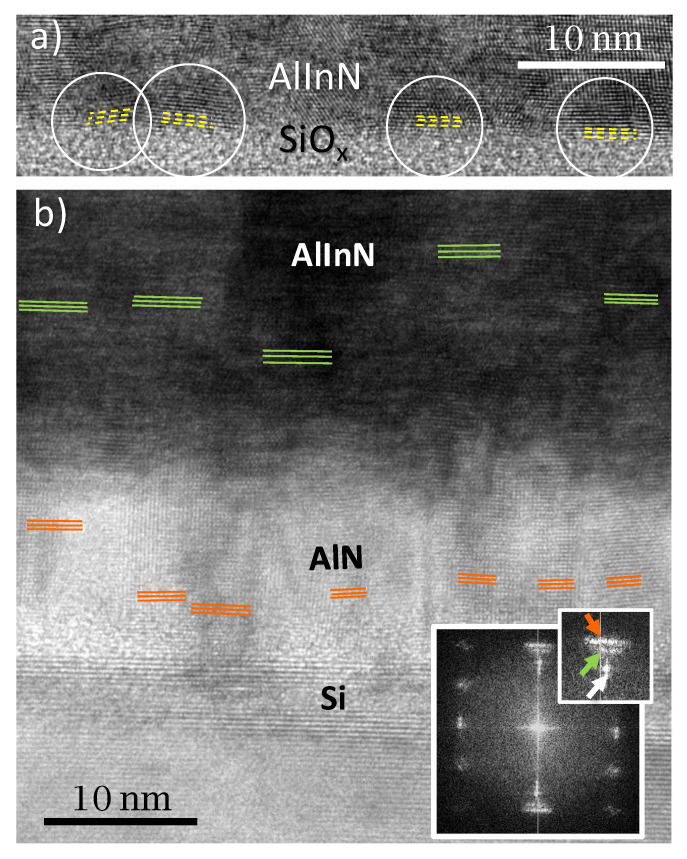
HRTEM images showing the misaligned crystal domains on (**a**) AlInN grown on bare silicon and (**b**) AlInN grown on 15 nm-thick AlN, along with their FFT (inset), showing the growth plane of AlN (orange), AlInN (green), and Si (white).

A study of the interface shows misaligned domains on the AlInN layer near the interface when it was grown directly on the Si substrate (see the yellow lines in Figure 6a). This effect can also be seen in the sample grown using the AlN buffer layer, but, in this case, the effect is weaker in the AlInN layer grown on top, while it is stronger within the AlN layer (see Figure 6b, where orange and green lines point to the growth plane orientation of neighboring grains. The FFT, included in the inset, shows elongated Bragg reflections for AlN (orange) and AlInN (green) as consequence of the misalignment). Thus, the introduction of an AlN buffer layer reduces the grain misalignment on the AlInN layer through the accommodation of misaligned crystal domains already in the AlN buffer, in such way that the effect is attenuated through the interface with the AlInN. This mechanism allows for the improvement of the crystal quality of the AlInN layer through a reduction of the misalignment of neighboring crystal domains and grain boundaries.

## 4. Conclusions

In this work, it was demonstrated that the inclusion of a 15 nm-thick AlN buffer layer improves the crystalline quality of the subsequent 80 nm-thick AlInN layer, when it is grown on p-Si (111) by RF sputtering. This was confirmed by the reduction of the FWHM of the (0002) AlInN rocking curve from 5.6 to 5.3°. Additionally, the TEM studies performed showed that the AlN buffer layer leads to a reduction of one order of magnitude of the density of defects and to the elimination of the amorphous (SiO_x_N_y_) interface, thanks to the high kinetic energy of the species during the buffer growth. Moreover, the AlN buffer layer acts as a barrier against oxygen diffusion into the AlInN layer.

The analyses reveal an epitaxial relationship between the AlInN and AlN layers with the silicon substrate, which is 90° in-plane rotated from the most reported layer in the system, which is the following: (0001)[11–20] AlInN||(0001)[11–20] AlN||(11-1)[112] Si. 

## Data Availability

Data is contained within the article.
